# Plan for Regulating the Practice of Physic

**Published:** 1806-10

**Authors:** 


*Soho Square, August* 9, 1806.

At a numerous meeting of the Faculty, held this evening at the house of the Right Hon. Sir Joseph Banks, Bart. K. B. President of the Royal Society, &c. Dr. Harrison laid upon the table the Answers he had received to the different circular letters transmitted to the public bodies and individual practitioners of the united kingdom, in pursuance of a former Resolution. He then presented the following Plan for better regulating the Practice of Physic in its different branches; which being read and considered, the subsequent Resolutions were entered into.

### PLAN.

1st. "That no person shall practise as Physician, unless he be a graduate of some University in the United Kingdom,

Kingdom, and has attained the age of twenty-four years. That he shall have studied the different branches of Physic in an university, or other respectable school or schools of Physic, during the space of five years, two of which shall have been passed in the university where he takes his degree.

2dly, "That no person shall practise as Surgeon under three and twenty years of age, nor until he has obtained a diploma or licence from some one of the royal colleges of surgeons or other chirurgical corporations of the united kingdom.—That he shall have served an apprenticeship of five years to a practitioner in surgery, and afterwards have spent at least two years in the study of Anatomy and Surgery in a reputable school or schools of Physic.

3dly, "That no person shall practise as an Apothecary until he shall have served an apprenticeship of five years to some regular Apothecary, or Surgeon practising as an Apothecary;—that he shall have studied the different branches of Physic in some reputable school or schools during the space of at least one year, and shall have attained the age of twenty-one years.

4thly, "That no man shall practise Midwifery, unless he has attended anatomical lectures twelve months, and received instructions for the same term from some experienced Accoucheur, and shall have assisted at real labours.—And that no female shall practise Midwifery without a certificate of fitness and qualification from some regular practitioner or practitioners in that branch.

5thly, "That no person shall follow the business of a retail Chemist or Druggist, unless he shall have served an apprenticeship of five years to that art.

6thly, "That none of these restrictions be construed to affect persons at present regularly practising in the different branches of medicine.

7thly, "Whether Physicians shall be entitled to recover their fees by the usual legal means?

8thly, "That a register shall be kept of all Medical Practitioners in the United Kingdom, and every person in future entering upon the practice of any branch of the profession shall pay a fine on admission, the amount and disposition of which to be settled and specified hereafter.

---

Resolved,

1st. That it appears from the returns to the circular letters, that the abuses complained of do exist to a great degree in every part of the United Kingdom; and that the necessity necessity for adopting regulations for their correction is universally admitted.

2dly, That it seems to be expedient that the plan proposed by Dr, Harrison be adopted as the basis of regulation; subject, however, to such alterations as may hereafter appear to be necessary.

3dly, That Sir Joseph Banks and Dr. Harrison be requested to wait again upon the Right Honourable Lord Henry Petty, to state to him the progress of the undertaking, and to consult him upon further measures.

4thly, That the following gentlemen be appointed a committee to confer and correspond with the different public bodies of the United Kingdom, upon the subject of the proposed regulations; — that they be requested o report their proceedings from time to time, and to take such other steps as they may judge necessary. Names of the committee: Sir John M. Hayes, Bart.; Sir Walter Farquhar, Bart.; Doctors Blackburn, Harrison, Garthshore, Fearson, Stanger, Willan, Clutterbuck, and Secretary.

5thly, That a voluntary subscription of One Guinea each be received from the town and country practitioners, by any member of the committee, to enable them to prosecute the important objects in which they are engaged. (The names of subscribers to be published hereafter.)

6thly, That Dr. Harrison be requested to circulate the above Plan and Resolutions of this evening among the Faculty of the United Kingdom, in the manner of the former circular letter.

7thly, That since persons of every rank and occupation in life are deeply interested in the proposed regulations, the Faculty are particularly requested to submit them to the principal inhabitants of their respective districts, by convening meetings, or in any other mode which they may think proper.

8thly, That the thanks of this meeting be given to the Right Hon. Sir Joseph Banks, for his continued attention to the Association, and the important objects of their pursuit.

